# Barriers and facilitators of return to work for loss of income claimants: Healthcare workers’ perspectives

**DOI:** 10.4102/ajod.v14i0.1442

**Published:** 2025-06-04

**Authors:** Gofaone L. Modise, Catharina J.E. Uys, Eileen du Plooy

**Affiliations:** 1 Department of Occupational Therapy, Faculty of Health Sciences, University of Pretoria, Pretoria, South Africa; 2 Case Management, Motor Vehicle Accident Fund Botswana, Gaborone, Botswana; 3 Claims Department, Botswana Medical Aid Fund, Gaborone, Botswana; 4 eFundanathi, Faculty of Health Sciences, University of the Witwatersrand, Johannesburg, South Africa

**Keywords:** barriers, facilitators, return to work, claims disability management, road traffic accident injuries, case management

## Abstract

**Background:**

Road traffic accidents (RTAs) are a global and public health concern affecting a third of the world’s population mainly in low- to middle-income countries, particularly affecting young people. Returning to work (RTW) following an RTA is essential for better health and financial outcomes. The motor vehicle accident (MVA) Fund Botswana assists loss-of-income (LOI) claimants with medical assistance, compensates for loss, advocates and facilitates RTW.

**Objectives:**

The study aims to identify barriers to and facilitators of RTW for LOI claimants as experienced by health care workers (HCWs).

**Method:**

A qualitative explorative design included six healthcare workers who had worked with the MVA Fund on RTW for at least 5 years through purposive sampling. Data collection was done using a focus group discussion. Thematic analysis was conducted using Atlas.ti, with data interpretation guided by the ecological case management model.

**Results:**

The main themes were healthcare systems, legislation and insurance systems, personal and workplace systems, which were further classified into eight subthemes relating to barriers and facilitators.

Barriers included ineffective case management and how claimants perceived work. Facilitators included clear insurance RTW guidelines and workplace support and education level.

**Conclusion:**

Successful RTW can be achieved through multidisciplinary collaboration of HCWs. While legal and healthcare systems play vital roles in RTW, personal factors and workplace systems cannot be ignored.

**Contribution:**

Understanding the barriers to and facilitators of RTW will assist in implementing RTW interventions to improve patient outcomes, health, livelihoods, quality of life and guide RTW operations to ensure a coordinated process in the insurance industry in Africa.

## Introduction

### Background

Occupation is recognised as a fundamental aspect of human life, encompassing the meaningful and purposeful activities that individuals engage in as part of their daily existence. It extends beyond mere tasks or work to include all forms of doing that contribute to well-being, identity and social participation. Curtin et al. (2009) stated that an occupation must possess five key characteristics: it must be active, purposeful, meaningful, contextualised and human (Curtin et al. 2009). Similarly, Baum, Christiansen and Bass (2024) define occupations as what we do, emphasising that they form the basis of how we feel about ourselves and how we perceive our identity (Baum et al. 2024). According to the World Federation of Occupational Therapists (WFOT), the aim of occupational therapy is to:

… promote, develop, restore, and maintain abilities needed to cope with daily activities to prevent dysfunction. Programs are designed to facilitate maximum use of function to meet the demands of the person’s working, social, personal, and domestic environment. (www.WFOT.org)

Therefore, occupational therapists (OTs) play a vital role in enabling individuals to engage in meaningful occupations, particularly after an injury or life-altering event. This is equally important for injured workers, as engaging in work has been shown to contribute positively to health and overall life satisfaction (Soeker 2014).

Reintegration into the workplace is a priority for stakeholders following an injury or sickness. Return to work (RTW) is an effort to enable the workplace reintegration of an employee following absence after a sickness or injury (American Occupational Therapy Association 2021; Whiteford et al. 2018; Wilcock 2006). Working contributes to positive health and life satisfaction, among other benefits (Cancelliere et al. 2016; Soeker 2014; Wilcock 2006). Being unable to work because of illness will result in considerable costs because of disability, sickness, absence and loss of productivity. Any disability arising from road traffic injuries (RTIs) is not only a health issue but may impact participation in social activities and working life (Abedi et al. 2022; Loisel, Anema & Anema 2013). Similarly, employers bear excessive costs of hiring replacements when injured employees have not returned to work. Insurance companies also suffer economic losses from work disabilities and welfare pay-outs. Therefore, RTW is of economic benefit and interest to all stakeholders (Figueredo et al. 2020).

Road traffic accidents (RTAs) are a growing public health concern; they burden healthcare systems (HCSs) and the individuals who suffer a loss of income (LOI) because of them. They affect not only those who encounter RTA but also the economies of the countries affected. The RTW process following RTAs requires prompt collaboration between injured employees, health care workers (HCWs), employers and funders or compensation boards (Cancelliere et al. 2016; Collie et al. 2019; Giummarra et al. 2017; Pelissier et al. 2017). Effective and well-coordinated RTW programmes are positively associated with successful RTW (Gane et al. 2019). However, the increase in RTAs, their socioeconomic impacts and the need for disability benefits threaten the sustainability of social security agencies such as the motor vehicle accident (MVA) Fund Botswana that invest in RTW programmes (Stefan et al. 2012).

For vulnerable populations, such as people from low economic backgrounds and people with disabilities, failure to RTW may exacerbate their poverty (Jain et al. 2020; Kamdar et al. 2020). Early RTW is paramount for people in low-income brackets as their loss of employment or income compromises them further. The MVA Fund Botswana compensates victims of RTAs and implements RTW programmes for these individuals. Road traffic accidents are a leading cause of morbidity and death worldwide, especially in low- to middle-income countries (LMICs) (World Health Organization 2020). Botswana’s fatality rate (20.1 per 100 000/year) remains higher than the global rate (17.4 per 100 000/year) and continues on an upward trajectory which is of concern (Juillard et al. 2010; Kenardy et al. 2014; Munuhwa et al. 2020; Mwandri & Hardcastle 2018; World Health Organization 2020, 2023). In Botswana, RTAs constitute a significant cause of mortality and morbidity, with 68% of the deaths being preventable (Motsumi et al. 2020). The frequency of RTAs, inadequate emergency and healthcare services increases medically preventable deaths and disabilities, experienced in sub-Saharan Africa (SSA) and most LMICs (Chatukuta 2020; Motsumi et al. 2020, 2022).

The MVA Fund Botswana supports claimants with medical undertakings that cover assistive devices and quality-of-life enhancements, among other benefits. In addition, the claimants receive RTW advocacy, with those losing their income being eligible for a LOI benefit. The LOI benefit is payable upon assessment and capped at BWP6000 ($520.29) per month or the prevailing minimum wage rate for the period of incapacitation (Motor Vehicle Accident Fund Botswana 2018). The MVA Fund Botswana must facilitate RTW programmes for such claimants where HCW providers are central to the facilitation of the RTW programme both at facilities and internal to the MVA Fund through the case management department. Case management is defined as a:

[*C*]ollaborative process of assessment, planning, facilitation, care co-ordination, evaluation and advocacy for options and services to meet an individual’s and family’s comprehensive health needs through communication and available resources to promote quality cost-effective outcomes. (Case Management Society of America 2018) (p.6)

Case management at MVA Fund Botswana is intended to support claimants injured in RTAs through medical and rehabilitative assistance to improve their chances of attaining optimal functioning and gaining independence. The Fund collaborates with medical professionals, health facilities and family members of the injured in the claimant’s rehabilitation process (MVA Fund Botswana 2020). Where an RTA occurs, the Fund pays for the cost of care provided by the HCWs, from emergency medical services to rehabilitation and RTW. Health care workers determine the cost and duration of the benefit through either sick leave, a Functional Capacity Evaluation (FCE) or occupational medicine assessment outcomes. Where advocacy can be held for reasonable accommodation at the claimant’s workplace, the HCWs lead the advocacy through the RTW programme. Through this process, the Fund manages the claimant’s rehabilitation journey and engages with all relevant stakeholders such as the claimant’s employer to return them to work (Figueredo et al. 2020). Returning claimants to work also directly benefits the Fund’s financial sustainability when the LOI payout ceases and produces an active member of the community. Returning claimants to work is also a benefit to the employers as they retain their talent and resource and do not incur expenses related to recruiting and training replacement employees (MVA Fund Botswana 2020).

Therefore, claimants’ lived experiences are vital in informing funders of interventions and what possible barriers and facilitators to RTW could be. The relationship between HCWs’ responsibilities, the duration of compensation claims and their role in facilitating RTW remains poorly understood (Ng et al. 2021). Although RTW barriers and facilitators are well known, information about RTW following RTAs in LIMC or SSA is lacking. In addition, RTW also affects workers injured at work who may need to be facilitated to RTW. Our qualitative study investigated barriers to and facilitators of RTW for LOI claimants through the lens of the HCWs’ experiences and is a continuation of gaining a deeper understanding of the problems claimants experience in RTW within HCSs.

## Theoretical framework

**FIGURE 1:**
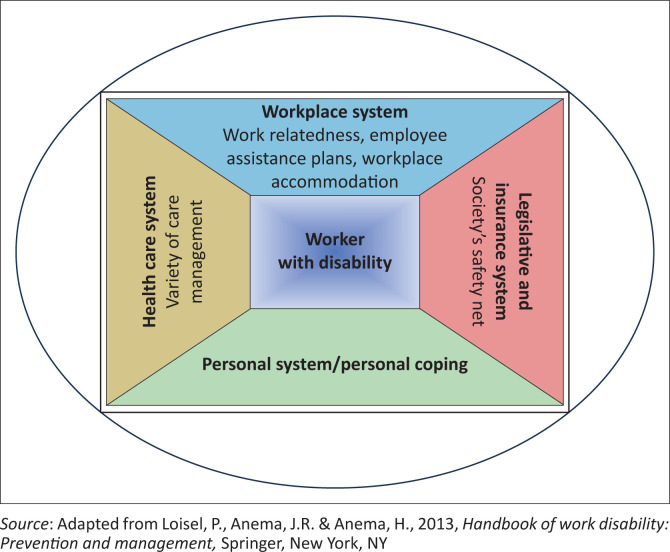
Ecological case management model of return to work.

The ecological case management model and workplace arena for disability primarily focuses on the perspectives of all stakeholders (Loisel et al. 2013). The model conceptualises RTW in addition to personal systems determined by complex interactions between the workplace, disability payers, insurance carriers and HCWs. The arena (Figure 1) appreciates the injured worker at the centre of the system, influencing their RTW.

The model identifies the following four main themes that are used for deductive analysis: the HCS; the Legislation and Insurance System (LIS), encompassing the compensation system with its local regulations and actors; the Personal System (PS), encompassing social relationships and, last, the Workplace System (WPS), with its main sociotechnical structures. Healthcare workers therefore play a vital role as part of the HCSs and are often a link of care and advocacy towards the claimant, the WPSs and the legislation and insurance system.

## Methodology

### Study design and setting

A qualitative, explorative design, using a single focus group discussion, allowed the first author to build a robust understanding of what HCWs experience as barriers and facilitators of claimants’ RTW through the lens of HCWs experiences who worked at the MVA Fund Botswana.

Our study was conducted within the MVA Fund organisation in Gaborone, Botswana. The authors had access to the Fund’s database and the HCWs who participated in the RTW programme. Although the focus group discussion was held in Gaborone, the HCWs support claimants from all over Botswana.

### Study population and sample

The population of interest was HCWs who worked with LOI claimants on the RTW programme in the MVA Fund Botswana. The sampled HCWs would have worked closely with the claimants on their RTW journey through the MVA Fund Botswana’s RTW programme. The experiences of HCWs having worked with claimants in their effort to return them to work from the acute stages, through rehabilitation and ultimately RTW, are not known. Six multidisciplinary HCWs with at least 5 years’ experience at the MVA Fund RTW programme were purposively sampled based on their knowledge and experience. The HCWs would have been engaged by the MVA Fund through their different institutions whether public or private as a part of the RTW programme from January 2015 to December 2020. An email was sent to all the HCWs who were on the MVA Fund database and met the inclusion criteria. Once they acceded to the request or showed interest, HCWs were recruited via email, and a convenient date and venue for the focus group discussion was arranged for those who accepted the request.

### Data collection and organisation

Information explaining our study was shared before the focus group discussion, and written informed consent was sought prior to the focus group discussion. The focus group was conducted mainly in English, but participants were encouraged to engage in the most comfortable language (Setswana or English) throughout the 2-h discussion. The focus group discussion was audio recorded and saved on the first author’s password-protected laptop to enhance confidentiality. The recordings were then transcribed verbatim (Leavy 2017) by a trained bilingual research assistant, who is trained in research methodology. A second bilingual research assistant who is also trained in research methodology translated the transcriptions into English and verified the correctness of the translations against the audio recordings. The translated scripts were member checked by one of the focus group discussion participants, and the correctness of the translations was verified, thereby ensuring transferability. The above steps ensured the rigour of the study. To enhance credibility, the first author used probing questions to sustain deeper engagement with the HCWs and triangulation of sources among the HCWs to gain a deeper understanding of some of the statements made (Lincoln & Guba 2013). The focus group lasted almost 2 h until saturation was reached. Records were meticulously kept enhancing authenticity, with a clear decision trail, ensuring consistency and transparency. To enhance dependability, the first author engaged the second and third authors to debrief and review the inquiry process and the data. The whole team interpreted and recommended findings to ensure consistency. The first author kept a reflective journal to reduce bias having worked at the organisation that was being researched (Lincoln & Guba 2013).

### Ethical considerations

Ethical clearance to conduct this study was obtained from the University of Pretoria Faculty of Health Sciences Research Ethics Commitee (No. 255/2021). Informed and written consent was also sought from the MVA Fund Botswana’s Chief Executive Officer (CEO). The Ministry of Health Botswana Research Unit further issued a study permit for the research (No. HRDP:6/14/1). All participants provided written consent and an oral consent to be audit recorded.

### Data collection materials

The Focus Group Discussion (FGD) questions explored HCWs’ experiences working on the RTW process with MVA Fund claimants and whether the return-to-work programme was coordinated effectively and documented to meet stakeholders’ expectations. The HCWs were initially asked to share from their experiences what they believe made RTW easier or more difficult for LOI claimants at MVA Fund Botswana. They were further asked from their experiences if the LOI benefit made it easier or difficult for the LOI claimants to RTW. Finally, they were then asked what they imagined could be done to improve the RTW process and programme. These were done with probing for statements in which the first author needed further elaborations. A pilot study was conducted before the focus group discussion with a sample of three HCWs who were not part of the study but matched the inclusion criteria and had experience working on the RTW programme to gauge their reception of the questions. The questions were further refined with the second and third authors for credibility and trustworthiness to ensure that they met the study’s aims and to reduce bias from the first author who had worked at the organisation. This was done to verify whether respondents would easily understand the questions (Creswell & Poth 2018). Subsequently, the FGD questions were revised to improve their understanding and repeatability.

### Data analysis

The authors employed a hybrid thematic analysis, integrating both inductive and deductive reasoning. This mixed approach highlights the value of combining theory-driven analysis with data-driven insights, ensuring that the ‘voices of the participants are valued, while simultaneously allowing for more theory-led analysis’ (Proudfoot 2023). Other researchers have supported this approach, which involves the use of pre-determined themes, derived from an established theoretical framework – in this case the ecological case management model. This data analysis framework was developed through engagement with existing evidence and the MVA Fund case management setting, representing a deductive component of the analysis (Proudfoot 2023). At the same time, the inductive component involved the generation of themes directly from the data. The sub-themes emerged organically from the data, complementing and mutually enhancing the predefined themes. Data analysis was conducted using Braun and Clarke’s thematic analysis approach (Braun, Clarke & Gray 2017). The author applied deductive coding to categorise the main themes and used inductive reasoning to identify sub-themes. Although Braun and Clarke’s six-phase process for thematic analysis was used as a guiding framework, it was adapted in our study because of the predefined theoretical framework – the ecological case management model, which structured the analysis into four main themes (Braun et al. 2017).

**Phase 1 Familiarisation with the data:** The authors familiarised themselves with the data before uploading them to Atlas.ti 25, a qualitative data analysis software that supports researchers in conducting thematic analysis as outlined by Braun and Clarke (Atlas.ti 2024).

**Phase 2 Generating initial codes:** After familiarising ourselves with the data, we proceeded to generate initial codes within Atlas.ti. These codes represented meaningful smaller components of the research topic with relevant text coded according to the research question, ‘what are the barriers and facilitators of RTW and what made it easier or difficult for the claimants to return to work?’. They identify and categorise important features of the data, which lays the foundation of emerging themes. This is vital in ensuring the data are codified; the authors had to pay attention to detail and do this using a structured approach.

**Phases 3 and 5 (integrated):** The generated codes were then categorised into the four primary themes of the ecological case management model, as proposed by Loisel et al. (2013): HCSs, personal systems, WPSs and legal and insurance systems (Loisel et al. 2013).

**Phase 4 Reviewing themes:** Given the diversity of the ideas emerging from the data, subthemes were generated through inductive reasoning where themes are developed directly from the data, without imposing a pre-existing framework or theory. This approach allows themes to emerge naturally, capturing the depth and complexity of the dataset. For our study, inductive analysis was particularly valuable as the predefined ecological case management categories could not fully accommodate the emerging meanings within our data. As a result, the following are subthemes of the ecological model: (1) *Workplace systems* – External Environment, Organisational, Department, Job positions; (2) *Healthcare systems* – Interdisciplinary and Inter-organisational Team, Multi-disciplinary team, other healthcare profession, attending physician; (3) *Personal systems* – Physical, Cognitive, Affective and Social Relationships; and (4) *Legal and insurance systems* – Compensation agent, Insurer Case worker, Regulations of jurisdictions, Provincial or federal laws (Atlas.ti 2024).

**Phase 6 Categorising barriers and facilitators:** The inductive subthemes were then categorised into barriers and facilitators for HCWs to recognise and implement following the dissemination of findings. Each sub-code was generated based on the unique characteristics and qualities emerging from the data as quoted from the participants and not pre-conceived notions.

**Phase 7 Finalising themes through iteration**: The iterative process included merging overlapping subthemes, particularly those with lower frequency, subdividing broad themes into more precise subthemes and eliminating themes that lacked sufficient supporting evidence. Through this process, the authors ensured that the final set of themes accurately reflected the data, capturing the richness and complexity of the studied phenomenon. The resulting thematic structure provided a comprehensive understanding of the barriers and facilitators of RTW (Atlas.ti 2024).

## Results

The six healthcare workers who participated in the focus group discussion were mainly rehabilitation personnel who had participated in the MVA Fund Botswana’s RTW programme for at least 5 years. The sample, as per Table 1, included four occupational therapists and two physiotherapists (PTs), three males and three females. These were made up from practitioners in the public sector, the parastatal, academia and private practice. The sample has general experience of the different sectors, how they have experienced working with MVA Fund Botswana and their experiences in barriers and facilitators of RTW for the claimants they have worked with on the RTW programme. Because of the limited population of allied healthcare in Botswana, especially occupational therapists, the demographic details are limited to their years of experience and the sector in which they work, as the authors did not want to compromise the identity of those who participated in our study.

**TABLE 1:** Socio-demographics of participants in the FGD.

Participant	Profession	Place of work	Experience
P1	Occupational therapist	Parastatal	Over 5 years
P2	Occupational therapists	Private	Over 10 years
P3	Occupational therapist	Government	Over 10 years
P4	Physiotherapist	Academia	Over 10 years
P5	Physiotherapist	Private sector	Over 5 years
P6	Occupational therapists	Private practice	Over 5 years

FGD, focus group discussion.

The four themes from the ecological case management model were used for deductive reasoning. Inductive reasoning generated 18 subthemes (Table 2). Of these 18 subthemes, 10 were facilitators of RTW, while eight were barriers.

**TABLE 2:** Themes, subthemes and the frequency of occurrence.

Themes	Subthemes	Frequency
**Healthcare system**		
Barriers	Ineffective case management	50
	Claimants’ motivation for financial compensation	13
	Overservicing by providers	9
	Lack of knowledge and awareness of the fund products and services	19
Facilitators	Early medical and RTW intervention	6
	Positive interdisciplinary collaboration	11
**Legislation and insurance system**		
Barriers	Lack of robust return to work regulatory framework	28
Facilitators	Effective regional, national and international RTW laws or policies	24
	Clear compensation agency processes for both medical intervention and RTW program	26
**Personal system**		
Barriers	Claimants’ culture and perception of the value of work	25
	Heavy and manual job or occupation type	16
	Lack of social networks, social status and intrinsic motive	6
Facilitators	Motivation	9
	Positive social status, networks and support	29
**Workplace system**		
Barriers	Lack of workplace support and reasonable accommodation	8
Facilitators	Effective return to work case management	8
	Workplace support and reasonable accommodation	12
	High educational level	12

RTW, returning to work.

## Healthcare systems

All HCWs are expected to ensure universal health coverage. For the HCSs, the most common barrier was a lack of effective case management (Table 2), followed by a lack of knowledge of the fund process and motivation for financial compensation by claimants. The lowest in terms of frequency was the overserving that the HCWs had experienced from their colleagues that kept the claimants in care for longer than necessary delaying RTW. Health care workers felt that the MVA Fund case management was ineffective in terms of processes that affected turnaround times and deliverables for RTW for both claimants and the providers as they were unsure of outcomes of their requests. Health care workers described how professionals had limited knowledge of the fund’s processes, which limited their advocacy and, at times, interventions and referral pathways.

### Healthcare system barriers

When asked if they had signed a formal RTW agreement with the claimants, employer and/or MVA Fund Botswana during the RTW programme they worked on, none of the HCWs had ever signed a RTW plan with employers, funders or claimants. This was despite having similar agreements with other organisations they were involved with for RTW programmes. There was no formal guideline for compiling a RTW plan; they indicated that each HCW was doing what they deemed proper, which caused much confusion regarding expectations for employers, claimants and funders. Other HCWs did not know about specific services that could accelerate RTW or were unaware of their role in ensuring that the necessary resources are requested from the fund, such as previous medical records.

In relating their experience, one participant echoed the lack of structure and effective case management, Participant 2 lamented the lack of coordination of care and silo mentality in some healthcare facilities:

‘… a patient goes to Hospital x for 3 weeks … 5 weeks … at Hospital x and there’s no OT (occupational therapist), there’s no one. And then two years later take them to OT. OTx is alone and she’s not collaborating with anyone, there’s no teamwork on this patient. So, how do you expect outcomes from this patient. That is why you end up with a myriad of problems leading from one to the other one to other …’ (P2, OT private practice, over 10 years experience)

Another participant echoed that the lack of effective RTW may be because RTW is deemed an afterthought, while participants believed that some of the barriers emanated from the MVA Fund itself because of a lack of structure in the RTW process:

‘… I think the challenges that I find let’s start from MVA Fund is that one- case managing of these issues, … out of nowhere eh a client will be referred to you. … maybe an employer was crying foul somewhere crying about – what is happening with this patient? I’m about to fire them- I’m about to relieve them from work and then all of a sudden MVA starts saying yes, we need to do something about it. And then when they come to you realise that aah but this person should have long been there, it’s after 3 years they come to you and expect you to do some return to work. I mean the employer has moved and you feel pity for the employer because umm they have uh they are production for instance especially most employers who are production oriented that they are not just a social service but the production oriented client or business.’ (P6, OT private practice, over 5 years experience)

‘Hey, it’s just tiring even to deal with MVA in things like that. So, it is not well structured …’ (P3, OT government, over 10 years experience)

### Healthcare system facilitators

Health care workers identified two HCSs facilitators, namely, early intervention and interdisciplinary collaboration. The HCWs explained that when they intervened early and had all the information and collaborated among themselves and with the MVA Fund and employers, the claimants were more likely to RTW:

‘[*W*]here there was success … is … when we was working … together with MVA – service provider, MVA and then engaging the employer and then advocating … It could mean training the employer about whatever disability that there was … educating them on the issues … they could do job modification. They could do a number of things to facilitate to help this client go return to work. … I found them gore uh they were successful when we used to work as a group … as a team.’ (P6, OT private practice, over 5 years experience)

‘… the next thing is the collaboration that has been over emphasised. And it’s time framed. It’s they will say we want to know, in 6 weeks you say you are doing work conditioning or work hardening how far can you go in 6 weeks. after that time you sit down and review. there’s a lot of follow up in that as well … it’s the structure that works …’ (P2, OT private practice, over 10 years experience)

## Legislation and insurance systems

Facilitators of RTW were effective, namely regional, national and international RTW laws or policies and transparent compensation agency processes for both medical intervention and RTW programmes. Health care workers felt that RTW could be easier if some of the legislation-mandated RTW policies and if the MVA Fund processes were clear on how far they can assist the client.

### Legislation barriers

According to the LIS theme, the most recurring barrier was the lack of an effective robust RTW regulatory framework. A robust RTW regulatory framework would drive much of what should happen on the ground, which can be cascaded to the national or organisational level. A robust framework will also negate the impact of occupational injustice. As Botswana works towards redefining disability laws, RTW national legislation and the United Nations Charter on the Rights of Persons with Disabilities (UN-CRPD) ratification are on the agenda. The HCWs deemed the lack of RTW legislation a barrier to RTW, as employees were not protected, and at times their advocacy efforts did not contribute much to RTW without the law on their side. They found this challenging in their roles as advocates for the claimants:

‘Some issues I think are system issues are originating from within the fund itself- a lack of uh a proper structure if any, a lack of a return-to-work policy at a government level. The fund doesn’t have a clear policy as to when can you really remove someone from the books, if they haven’t been compliant so there’s nothing really that hold them accountable. So that should be clearly articulated.’ (P4, PT academia, over 10 years experience)

‘[*W*]ho is really supposed to coordinate between the employer and the client and the claimant and the therapist.’ (P2, OT private practice, over 10 years experience)

‘But there’s no plan written.’ (P1, OT parastatal organisation, over 5 years experience)

‘I think uh the fund and it’s not because the fund doesn’t want but it’s because uh above the fund, we don’t have a regulatory framework as we were saying, a properly structured one. So there’s really a lot of loopholes that any of those are barriers to return to work.’ (P4, PT academia, over 10 years experience)

### Legislation facilitators

Only two facilitators were identified: transparent compensation agency processes for both medical intervention and RTW programmes, followed by effective regional, national and international RTW laws or policies. Legislation and the insurance system, as previously alluded to, offer some form of financial compensation in some instances and govern the rules of both compensation and RTW, which play vital roles in RTW. Therefore, when claimants are clear on the process, they are likely to move on and also to establish expectations for all stakeholders, right from medical claims to rehabilitation to LOI benefits. Health care workers found that people were likely to RTW when they realised that they had exhausted all possible assistance from the MVA Fund Botswana. When the stakeholders worked together, HCWs felt that the success rate in advocacy and RTW was high:

‘Success came when MVA Fund uh is fully engaged umm to the extent that sometimes the fund would even ask that you go to meet with an employer and the with the claimant also available and you’re able to have that dialogue, those cases are usually successful.’ (P6, OT private practice, over 5 years experience)

‘And now when we talk about MVA comparing it with other with other companies you find that some companies are more structured. The fund needs to develop a well-structured return to work case management process’ (P4, PT academia, over 10 years experience)

‘The issue that also MVA is a social protection, you see. This it’s an organisation that gets talked about at Kgotla meeting you know. Makes it very vulnerable … At the parliament at that level.’ (P6, OT private practice, over 5 years experience)

The HCWs recognise that some mandates are beyond the scope of the Fund; however, through legislation and other political platforms, a structure can be developed that can swiften the process of RTW.

## Personal system

Three personal system themes related to cultural barriers; how claimants perceived the value of work, followed by occupying a heavy or manual job type. Facilitators identified under this main theme were a positive social status, networks and support. Within this sub-theme was the motivation to work or RTW, especially for young people and the employee’s reputation and relationship with the employer.

### Personal system barriers

In the PS, there were three barriers in terms of the value of work, which varies by job or position type. Only two facilitators were identified, namely ‘positive social status, networks and support’:

‘Umm. You know what there is a degree of a culture of entitlement umm which just doesn’t plague umm people who are low earners or unemployed, some wonder … Should I do go for loss of employment or should I go back to work.’ (P4, PT academia, over 10 years experience)

‘They want the government to do this. It’s just the mentality of saying what can I get. (from the government welfare programmes) And if you can get it without doing anything. I think it is not to say that the low-level income people we are like. However, I think it is a certain understanding. They don’t understand that occupation on its own like he was saying it gives you purpose.’ (P3, OT government, over 10 years experience)

‘Because they think … I have had an accident, so MVA can do this and this for me. However, even if they do not know, they will push it to see if they can get it. Therefore, we have that … Batswana sometimes we don’t value uh value work. Umm. That work is not just about getting money. It’s about our- you know-whole wellbeing.’ (P3, OT government, over 10 years experience)

### Personal system facilitators

The most frequent subtheme under the personal systems’ facilitators was positive social status, networks and support. These factors often offer claimants better healthcare because of access to better healthcare services, better options with regard to redeployments and role modifications because of higher education training:

‘Those that are doing well financially … are motivated … because it affects … their livelihoods financially much more. You have an executive who is injured … has a business, they don’t want to get bucked down with. disability so they do the best they can they are a bit aggressive they are quite motivated, “And you find that the spouses … the wife in this case the money that they are receiving from MVA won’t be enough. We had eh a finance officer who was high ranking in the government and the money that he is going to get was almost nothing compared to his salary. he did not want to be pitiful … they had debts that needed to be paid and things like that …’ (P3, OT government, over 10 years experience)

‘And … Performance … promotion. So now her performance was low. now she is stressed that if I don’t perform, how much is my boss or the employer going to tolerate of me under performance. But for her she has options like you are saying, high income earning they have options. So, she was now considering career change. So instead of going back to that particular job she was now thinking I can go and do farming because she has a farm somewhere … So those are some of the things that affect yeah. The process of returning to work.’ (P5, PT private practice, over 5 years experience)

‘… okay my husband is not high-income high income but fractured his elbow, what I saw him do I’ve never saw any of my clients do. You know where he goes through therapy and rehab and he is told by orthopaedic specialist by his physiotherapists by the OT that this is as much as you are going to get with your arm … since April this year he has been working with no OT, no physio, no orthopaedic specialist to get his arm where he wanted it to. The type of motivation … Because he knows what he has to lose. And what he has to … I’m just talking about being intrinsically motivated. When you have something to lose its more.’ (P2, OT private practice, over 10 years experience)

## Workplace systems

Work demands, organisational factors and expectations affect RTW prospects, with studies indicating a direct link between physical work demands and work absenteeism, especially where there is a high work pace and problems with relationships with colleagues.

### Barriers and facilitators

Under the WPSs theme, two facilitators were workplace support and educational level, while the barriers were lack of workplace support and reasonable accommodation:

‘[*T*]hose that are doing well financially you know they are motivated because it affects their livelihoods financially much more. So, you have an executive who is injured who has a business, a cattle-post who has this going on and they don’t want to get bucked down with uh disability so they do the best they can they are a bit aggressive they are quite motivated.’ (P4, PT academia, over 10 years experience)

‘It frustrates the client and also the employer because they don’t know what to do but they need this person to go to work. And then the other thing that I have observed is looking at the class 9 employees that we are talking about. One example is a guy I was working with who was a combi driver, he was involved in an accident as a passenger not his combi. His combi was parked home. But immediately when he was involved in an accident he was taken to the hospital. The owner of the combi had to employ someone.’ (P5, PT private practice, over 5 years experience)

‘Company x is not a very structured employer like Company Y … those ones would be more advanced in terms of their systems on how to they have HR systems. So, it meant that those who I was mostly successful with were companies which were organised like Company Y. But these ones you will find where there is no structure or not organi2ed, they’ve really moved on and left the client.’ (P6, OT private practice, over 5 years experience)

‘[*Y*]ou’ve finished you have to there is no way to redeploy them to. Because where they are there’s only one job. So, the key thing is to go back to what other skills do they have.’ (P1, OT parastatal organisation, over 5 years experience)

Work demands, organisational factors and expectations may affect RTW prospects, with studies indicating a direct link between physical work demands and work absenteeism, especially where there is a fast work pace and relationship problems with colleagues. Employers’ willingness to return their workers to work also seems to positively influence disability duration and costs (Loisel et al. 2013).

## Discussion

Our study aimed to identify barriers and facilitators of RTW for MVA Fund LOI claimants following an RTA as experienced by the HCWs through an FGD. There were four barrier themes within the RTW ecological model: Healthcare system-barriers, Legal and Insurance systems barriers, Personal systems barriers and WPS barriers and four facilitator themes: Healthcare systems facilitators, Legal and insurance systems facilitators, Personal systems facilitators and WPSs facilitators. The most frequent barriers were observed under the HCSs theme, followed by the Personal systems theme. Facilitators were mainly noted under the Legal and Insurance systems and WPSs themes. These agree with previous studies despite such studies being from high-income countries.

The lack of coordination and structure of the RTW programme was the most prevalent barrier, which also cut across multi-disciplinary cross-function. There was no accountability from either party because of a lack of documentation on the expectation of the programme. There were no formally established responsibilities by either party, despite the HCWs having signed and agreed processes for RTW with other organisations they worked with and having appreciated the effectiveness of a structured documented process. Studies indicate that a well-structured and coordinated RTW programme increases the likelihood of RTW (Gane et al. 2019; Wilbanks & Ivankova 2015). Communication between all stakeholders, employers, employees, HCW and funders has proven to be critical in establishing everyone’s role and responsibilities and ensuring they are fulfilled to the benefit of the client’s RTW (Cancelliere et al. 2016).

The HCS plays a central role in delivering both preventive and curative care, encompassing the personnel who provide the care and the facilities where the care is administered and all HCWs are expected to ensure universal health coverage. Within the HCSs, a lack of communication between HCWs, funders and employers may negatively affect the likelihood of returning to work. Role clarity is vital especially in complex injuries, as a lack of clarity can bring confusion and conflict between the stakeholders (Kosny et al. 2018). Therefore, working in silos and in conflict will affect the client’s RTW as per our study’s findings. Recent studies have indicated that medical care provided through workers’ compensation tends to be more costly than care provided through the general health system (Hani et al. 2023). Studies have reported contradictory results regarding the effects of compensation on work absenteeism. Comparisons between studies are hampered by differences in legislation or insurance rules in different countries and states.

In addition to HCSs barriers and facilitators, Chatukuta (2020) indicated that in Namibia claimants who receive state care are often less served than are those served by the MVA Fund Namibia, as they have access to private care through their medical insurance, and being a state patient is viewed negatively by HCWs. Chatukuta (2020) also posits that government facilities usually have higher staff-to-patient ratios and longer waiting periods, thereby delaying access to care. Others such as Motsumi et al. support these findings (Motsumi et al. 2020, 2021) on the burden of trauma care in Botswana specifically. Some of what HCWs echo above about the presence or absence of certain specialties in other facilities resonate with the findings of Chatukuta (2020), which makes case managing the LOI claimant a challenge in Botswana. In our study, HCWs indicated that they were successful when all the stakeholders worked together, and there was a clear structure and coordination. Other studies support this notion (Cancelliere et al. 2016). Therefore structure, a coordinated, documented RTW programme supported by national policies and regulations with explicit role allocations, time frames and outcome measures can be a facilitator of RTW in LOI cases in Botswana.

Over and above, a high burden of trauma, lack of trauma specialists and general healthcare challenges are barriers for case managers and funders in Botswana. Trauma care in Botswana is inconsistent; therefore, its delivery poses challenges for practitioners as was echoed by the HCWs in our study. However, Botswana is a middle-income country with tremendous opportunities to improve trauma care. However, centralised trauma services have not yet been developed, and epidemiological trauma data are lacking (Cox et al. 2018). Road traffic accidents and a high trauma burden remain a concern for the SSA region (Chatukuta 2020; Sharma 2008). With regard to healthcare challenges as seen in South Africa and some of the SSA, the process of creating a health system that is responsive to a population with both disability and varying rehabilitation needs is inevitably compromised by mediating differences in opinions for the identification of focus areas for service provision and resource allocation (Lieketseng, Cloete & Mji 2017).

Legislation and policy are basic to the cost-effectiveness of health and occupational interventions. Policymakers are responsible for deciding whether to include an intervention or social welfare benefit in the basic benefit package that is financed by taxes or social security contributions (Loisel et al. 2013). In our study, the legal and insurance systems contributed directly to the factors that influence RTW. Compensation systems, legal and insurance processes are acknowledged to cause stress. They are associated with increased disability, anxiety, depressive symptoms and lower quality of life, with HCWs at times finding it difficult to comprehend their role within the compensation system, thereby compromising disability management and RTW (Collie et al. 2019). These were cited particularly where claims administration processes were not well defined or well coordinated. The HCW in Chatukuta (2020) research, a study of the MVA Fund Namibia, which operates similarly to the MVA Fund Botswana, raised concerns regarding the ‘lengthy and complicated’ process of registering with the MVA Fund Namibia, which often leads to access to benefits being compromised. The knowledge and awareness of the agency processes also facilitate RTW. However, the employment act in Botswana is silent on RTW advocacy, and therefore most employers are restricted to the sick leave policy, which is about 20 days for private companies and 3 months for government employees and up to 6 months before formal laying off is activated (Modise et al. 2023). This is short for any severely injured claimants, especially in a country where trauma care is not at its best or where early intervention is still a challenge. Therefore, the laws do not protect persons with complex injuries and long-term work absences or disabilities to enable RTW. Other disability models also support research arguing that administrative policies and processes can impede RTW (Collie et al. 2019). For this reason, a RTW operational framework is vital for addressing these shortcomings when the barriers and facilitators are known. Without knowledge of the barriers and factors affecting RTW in compensatory systems within the working population, as experienced by all concerned stakeholders, it is difficult to target interventions to reduce sickness absence and promote RTW (Labriola 2008). We identified that compensation guidelines and RTW case management processes should be clarified for HCWs and claimants to manage all stakeholder’s expectations, responsibilities and outcomes. Effective RTW processes have also been attributed to success in RTW (Gane et al. 2019); therefore, there is a need for the MVA Fund Botswana to document its RTW process, clearly define and document the process with the employer, HCW and client to derive outcomes from the programme.

The other findings regarding facilitators, which also came as recommendations were that MVA Fund policies, do not exist in solitude, but alongside other welfare or social security policies as set by the country. Therefore, these directly impact on delivery of the MVA Fund RTW programme as the existing laws of Botswana underpin it. The HCWs indicated the need for RTW programmes to be entrenched at a national policy level, which would enable RTW to not be an ‘afterthought’ as indicated by the participants but to be at the forefront of all rehabilitation programmes. The current National Policy on Care for Persons with disabilities was last reviewed in 1996 (Chichaya 2019); therefore, there is a need for the policy to be reviewed to entrench areas such as RTW, advocacy and implementation of such programmes for employers as the country ratified the United Nations’ Convention on the Rights of Persons with Disabilities (UNCRPD). Comparisons between studies are hampered by differences in legislation or insurance rules in different countries, when comparing the Australian and American settings, whose laws support RTW initiatives.

Effective and timely RTW programmes are critical for achieving successful outcomes for the injured employee and the employer, and established programmes are cited as helping pave the way for successful RTW (Wilbanks & Ivankova 2015). There is now substantial evidence that the policy and practices of the administering agency can significantly impact on the health of injured workers (Collie et al. 2019). Claimants benefit from an evidence-based framework that advocates for them and empowers them to understand the importance of engaging in an occupation compared to receiving compensation. Our findings show that without robust RTW policies, it will be difficult for the stakeholder to return claimants to work effectively.

Compensation can never replace the actual value of occupational engagement, purpose or meaningful engagement. Long-term unemployment because of disability is not only expensive but also leads to LOI, emotional trauma, reduced quality of life and higher mortality rates and social security and substantial societal costs (Sjobbema et al. 2018). Therefore, effective RTW programmes can contribute to people’s livelihoods, socioeconomic status and improved health outcomes. The HCWs in our study did not see themselves as the change agents, advocates or facilitators of policies. There was also a need for empowerment and awareness as some of the processes the HCWs were not aware of are on the Fund’s public domains.

Health care workers perceived the personal systems barriers emanating from how claimants valued work, followed by the heavy-duty type of job or position the claimant held. Only two facilitators were identified, namely ‘social networks and status’ and ‘motivation’. Personal characteristics such as age, intrinsic motivation and socioeconomic status have been indicated to play both positive and negative roles in RTW (Cartwright & Roach 2016; Giummarra et al. 2017; MacKenzie et al. 1998). In Botswana, LOI claimants receive compensation only if they are unable to work; therefore, for low-income earners, compensation may exceed their usual income, and people may not value their work or push hard to RTW especially if the funds being received are adequate for their day-to-day cost of living. Therefore, seeing LOI as an incentive to not RTW was mainly observed in low-income populations who would also use the RTA incidents to seek other welfare services provided by the government, thereby delaying RTW. This was a barrier to RTW and HCWs found it tiring dealing with such cases. Success was found in cases where motivation for RTW surpassed the motivation for LOI, mostly where the claimants stood a chance to lose or if their earnings surpassed the cap applied by MVA Fund Botswana. Modise et al. (2023) described the socio-demographic class of the MVA Fund Botswana LOI claimants as mostly low-income earners who are in the informal sector without much formal education (Modise et al. 2023). Therefore, for these earners, disabilities put excess pressure on already existing economic challenges, and they may find it hard to comply fully to rehabilitation plans. Eventually, this affects RTW negatively. As the country’s unemployment rate rises, it creates greater competition for employment (Tinta 2023) and a RTW barrier for low-income earners as they are easily replaceable (Modise et al. 2023). Health care workers felt that even with this much motivation and healthcare support, LOI claimants could not RTW if they had certain pre-accident occupations or job types where the injuries they suffered made it impossible to RTW even with support or modification (Khorshidi, Marembo & Aickelin 2019). Return to work was notably more complex for people with severe injuries and low education such as those in the informal sector. Such claimants had manual jobs, and their jobs did not allow for much movement or modification within their field of work (Ferdiana et al. 2021).

Work demands, accommodation, organisational factors and expectations affect RTW prospects, with studies indicating a direct link between employer and collegial relationships and RTW. This is because social support during recovery from an injury can increase motivation and a sense of inclusion (Noll, Mallows & Moran 2022). We indicated workplace support as a facilitator and a lack of it as a barrier. Employers’ willingness to return their workers to work also seems to positively influence disability duration and costs (Loisel et al. 2013), and this could be attributed to the workers’ work ethic before the injury. The type of work the person did prior to the RTA or educational level and social class has been also linked to being a predictor to RTW success (Cancelliere et al. 2016). Those who are highly educated have more options to move within the organisation or market while those in the informal sector or less educated have limited options, often leading to being released from work on medical grounds. In addition, those with a higher social class have more motivation towards rehabilitation as they understand what is at stake. Health care workers have recommended that the MVA Fund should be better structured to facilitate the RTW process, with objective measurable outcomes and accountability from the claimants, employers, HCWs and the Fund. In their experience, when there was structure and collaboration between all stakeholders, who were kept abreast of the claimant’s rehabilitation proress, that is when RTW has been successful. Health care workers have echoed that the lack of RTW policies at a national level makes it difficult to hold employers accountable or ensure the retention of vulnerable groups within the employment market. Although HCWs had instruments to measure RTW such as FCEs, there were no legal grounds for enforcing the employer to keep the claimant in work as the law did not recognise these if the sick leave period had elapsed for instance or to provide reasonable accommodation for the claimant. This idea is novel as it will inform policies and advocate for RTW, given this evidence, which is unique to Botswana.

### Limitations of our study

Our study was based only on the MVA Fund Botswana; therefore, the results may not be generalisable to other insurance industries or MVA Funders. As some healthcare workers were private service providers of the MVA Fund Botswana, they may have been biased in their response, fearing that their businesses may be affected. There was a lack of diversity of acute care providers, such as surgeons, in the sample; however, in the Botswana context, the post-discharge teams and rehabilitation personnel are often the ones facilitating RTW. From a pragmatic perspective, our results might be relevant in similar contextual environments, such as workman compensation schemes and funders, such as the MVA Fund eSwatini, MVA Fund Namibia and the Road Accident Fund South Africa, which often share best practices on similar processes.

## Conclusion

We recommend that medical professionals should overcome the problem of working in silos to resolve disability and compensation cases, which can be solved only through multi-professional collaboration on RTW. In the MVA Fund Botswana, RTW should be prioritised in terms of laws and policies, both national and international. Organisations such as the MVA Fund and other legal institutions must support employers and employees in the recovery journey. However, social standing, education levels and support at work and home are important facilitators for RTW. Socio-economic status and early intervention play a vital role in successfully completing the RTW programme. While the MVA Fund continues to advocate for claimants to be retained in work and provides support in the form of employer advocacy, paying out for both LOI and medical assistance, more efforts are needed from the regulatory bodies to entrench RTW on both the employers, HCW and individuals who find themselves in RTAs. Health care workers need to be empowered to be advocates of both the public and private systems for the betterment of the RTW process, and stringent measures are needed for all parties to account on their role and to have a framework of reference that can be applied nationally for RTW processes that are owned by all stakeholders.

## References

[CIT0001] Abedi, M., Gane, E., Aplin, T., Zerguine, H. & Johnston, V., 2022, ‘Barriers and facilitators associated with return to work following minor to serious road traffic musculoskeletal injuries: A systematic review’, *Journal of Occupational Rehabilitation* 32, 13–26. 10.1007/s10926-021-09994-3 34241769

[CIT0002] Akbarzadeh Khorshidi, H., Marembo, M. & Aickelin, U., 2019, ‘Predictors of return to work for occupational rehabilitation users in work-related injury insurance claims: Insights from mental health’, *Journal of Occupational Rehabilitation* 29, 740–753. 10.1007/s10926-019-09835-4 30874999

[CIT0003] American Occupational Therapy Association, 2021, ‘Occupational therapy scope of practice’, *The American Journal of Occupational Therapy* 75(Supplement_3), 7513410020. 10.5014/ajot.2021.75S3005 34989779

[CIT0004] Atlas.ti, 2024, ‘How to do thematic analysis step by step’, viewed 20 November 2024, from https://atlasti.com/guides/thematic-analysis/how-to-do-thematic-analysis-step-by-step-guide.

[CIT0005] Baum, C.M., Christiansen, C.H. & Bass, J.D., 2015, ‘The Person-Environment-Occupation- Performance (PEOP) model’, in C.H. Christiansen, C.M. Baum & J.D. Bass (eds.), *Occupational therapy: Performance, participation, and well-being*, 4th edn., SLACK Incorporated, Thorofare, NJ, pp. 47–55.

[CIT0006] Braun, V., Clarke, V. & Gray, D., 2017, *Collecting qualitative data : a practical guide to textual, media and virtual techniques*, Cambridge University Press, Cambridge.

[CIT0007] Cancelliere, C., Donovan, J., Stochkendahl, M.J., Biscardi, M., Ammendolia, C., Myburgh, C. et al., 2016, ‘Factors affecting return to work after injury or illness: best evidence synthesis of systematic reviews’, *Chiropractic & Manual Therapies* 24, 32. 10.1186/s12998-016-0113-z 27610218 PMC5015229

[CIT0008] Cartwright, A. & Roach, J., 2015, ‘Fraudulently claiming following a road traffic accident: A pilot study of UK residents’ attitudes’, *Psychiatry, Psychology and Law* 23, 446–461. 10.1080/13218719.2015.1080148

[CIT0009] Case Management Society of America, 2018, *Standards of practice*, Case Management Society of America, Little Rock, AR.

[CIT0010] Chatukuta, M., *Road traffic injuries in Namibia. A mixed methods study to analyse the trends in mortality and morbidity due to road crashes, and to investigate the long-term effects of road injuries*, Doctoral dissertation, University College London, London, UK.

[CIT0011] Chichaya, T., 2019, ‘Applying the occupational justice framework in disability policy analysis in Namibia’, *South African Journal of Occupational Therapy* 49(1), 19–25. 10.17159/2310-3833/2019/vol49n1a4

[CIT0012] Collie, A., Sheehan, L., Lane, T.J., Gray, S. & Grant, G., 2019, ‘Injured worker experiences of insurance claim processes and return to work: a national, cross-sectional study’, *BMC Public Health* 19, 927. 10.1186/s12889-019-7251-x 31291915 PMC6621963

[CIT0013] Cox, M., Becker, T.D. & Motsumi, M., 2018, ‘Head injury burden in a major referral hospital emergency centre in Botswana’, *African journal of emergency medicine* 8(3), 100–105. 30456157 10.1016/j.afjem.2018.02.003 PMC6223603

[CIT0014] Creswell, J.W. & Poth, C.N., 2018, *Qualitative inquiry & research design: Choosing among five approaches*, Sage, Thousand Oaks, CA.

[CIT0015] Curtin, M., Molineux, M. & Supyk, J., 2009, *Occupational therapy and physical dysfunction: enabling occupation*, Edinburgh, Churchill Livingstone.

[CIT0016] Ferdiana, A., Post, M.W.M., Bültmann, U. & Van Der Klink, J.J.L., 2021, ‘Barriers and facilitators for work and social participation among individuals with spinal cord injury in Indonesia’, *Spinal Cord* 59, 1079–1087. 10.1038/s41393-021-00624-6 33828246

[CIT0017] Figueredo, J.M., Garcia-Ael, C., Gragnano, A. & Topa, G., 2020, ‘Well-being at work after return to work (RTW): A systematic review’, *Int J Environ Res Public Health* 17, 7490. 10.3390/ijerph17207490 33076302 PMC7602369

[CIT0018] Gane, E.M., Smits, E.J., Brakenridge, C.L., Gangathimmaiah, V., Jagnoor, J., Cameron, I.D. et al., 2019, ‘Functional and employment outcomes following road traffic crashes in Queensland, Australia: Protocol for a prospective cohort study’, *Journal of Transport & Health* 15, 100678. 10.1016/j.jth.2019.100678

[CIT0019] Giummarra, M.J., Cameron, P.A., Ponsford, J., Ioannou, L., Gibson, S.J., Jennings, P.A. et al., 2017, ‘Return to work after traumatic injury: Increased work-related disability in injured persons receiving financial compensation is mediated by perceived injustice’, *Journal of Occupational Rehabilitation* 27, 173–185. 10.1007/s10926-016-9642-5 27150733

[CIT0020] Hani, U., Monk, S.H., Pfortmiller, D., Stanley, G., Kim, P.K., Bohl, M.A. et al., 2023, ‘Effect of workers’ compensation status on pain, disability, quality of life, and return to work after lumbar spine surgery: A 1-year propensity-matched analysis’, *Journal of Neurosurgery: Spine* 1, 1–11. 10.3171/2023.2.SPINE221341 36964725

[CIT0021] Jain, R., Budlender, J., Zizzamia, R. & Bassier, I., 2020, *The labor market and poverty impacts of covid-19 in South Africa, (No. 2020-14)*. Centre for the Study of African Economies, University of Oxford, Oxford.

[CIT0022] Juillard, C., Labinjo, M., Kobusingye, O. & Hyder, A.A., 2010, ‘Socioeconomic impact of road traffic injuries in West Africa: exploratory data from Nigeria’, *Injury Prevention* 16, 389–392. 10.1136/ip.2009.025825 20805620

[CIT0023] Kamdar, B.B., Suri, R., Suchyta, M.R., Digrande, K.F., Sherwood, K.D., Colantuoni, E. et al., 2020, ‘Return to work after critical illness: a systematic review and meta-analysis’, *Thorax* 75, 17–27. 10.1136/thoraxjnl-2019-213803 31704795 PMC7418481

[CIT0024] Kenardy, J., Heron-Delaney, M., Bellamy, N., Sterling, M. & Connelly, L., 2014, ‘The University of Queensland study of physical and psychological outcomes for claimants with minor and moderate injuries following a road traffic crash (UQ SuPPORT): Design and methods’, *European Journal of Psychotraumatology* 5(1), 22612. 10.3402/ejpt.v5.22612 PMC4009486 24799996

[CIT0025] Kosny, A., Lifshen, M., Yanar, B., Tonima, S., Maceachen, E., Furlan, A. et al., 2018, ‘The role of healthcare providers in return to work’, *International Journal of Disability Management* 13, e3. 10.1017/idm.2018.4

[CIT0026] Labriola, M., 2008, ‘Conceptual framework of sickness absence and return to work, focusing on both the individual and the contextual level’, *Work* 30, 377–387. 10.3233/WOR-2008-00708 18725701

[CIT0027] Leavy, P., 2017, *Research design : Quantitative, qualitative, mixed methods, arts-based, and community-based participatory research approaches*, The Guilford Press, New York, NY.

[CIT0028] Lieketseng, N., Cloete, L. & Mji, G., 2017, ‘The experiences and challenges faced by rehabilitation community service therapists within the South African Primary Healthcare health system’, *African Journal of Disability* 6, 1–11. 10.4102/ajod.v6i0.311 PMC5645568 29062760

[CIT0029] Lincoln, Y.S. & Guba, E.G., 2013, *The constructivist credo*, Left Coast Press, Inc., Walnut Creek, CA.

[CIT0030] Loisel, P., Anema, J.R. & Anema, H., 2013, *Handbook of work disability : Prevention and management*, Springer, New York, NY.

[CIT0031] Modise, G.L., Uys, P.K., Masenge, A. & Du Plooy, E., 2023, ‘Relationship between demographic characteristics and return-to-work for loss of income claimants at the Motor Vehicle Accident Fund, Botswana’, *Work* 77(4), 1101–1114. 10.3233/WOR-220712 37781840

[CIT0032] Motsumi, M.J., Ayane, G., Kwati, M., Panzirah-Mabaka, K. & Walsh, M., 2021, ‘Preventable deaths following road traffic collisions in Botswana: a retrospective review’, *Injury* 52, 2665–2671. 10.1016/j.injury.2021.04.020 33888332

[CIT0033] Motsumi, M.J., Chinyepi, N., Difela, K., Ngwako, K., Sentsho, M., Chilisa, U. et al., 2022, ‘Assessment of surgical care capacity at non-tertiary hospitals in Botswana’, *Annals of African Surgery* 19, 193–199. 10.4314/aas.v19i4.6

[CIT0034] Motsumi, M.J., Mashalla, Y., Sebego, M., Ho-Foster, A., Motshome, P., Mokokwe, L. et al., 2020, ‘Developing a trauma registry in a middle-income country – Botswana’, *African Journal of Emergency Medicine* 10, S29–S37. 10.1016/j.afjem.2020.06.011 33318899 PMC7723909

[CIT0035] Motor Vehicle Accident Fund Botswana, 2018, Motor Vehicle Accident Fund 2017 annual report, viewed 20 January 2020, from https://mvafund.bw/wp-content/uploads/2021/03/2017-MVA-ANNUAL-REPORT.pdf.

[CIT0036] Motor Vehicle Accident Fund Botswana, 2020, Annual report 2019: Behavioural change key to sustainable road safety, viewed 20 January 2020, from https://mvafund.bw/wp-content/uploads/2021/03/2019-Annual-Report.pdf.

[CIT0037] Munuhwa, S., Govere, E., Samuel, S. & Chiwira, O., 2020, ‘Managing road traffic accidents using a systems approach: Case of Botswana-empirical review’, *Journal of Economics and Sustainable Development* 10, 176–188.

[CIT0038] Mwandri, M.B. & Hardcastle, T.C., 2018, ‘Burden, characteristics and process of care among the pediatric and adult trauma patients in Botswana’s main hospitals’, *World Journal of Surgery* 42, 2321–2328. 10.1007/s00268-018-4528-7 29450701

[CIT0039] Ng, W., Slater, H., Starcevich, C., Wright, A., Mitchell, T. & Beales, D., 2021, ‘Barriers and enablers influencing healthcare professionals’ adoption of a biopsychosocial approach to musculoskeletal pain: A systematic review and qualitative evidence synthesis’, *Pain* 162, 2154–2185. 10.1097/j.pain.0000000000002217 33534357

[CIT0040] Noll, L., Mallows, A. & Moran, J., 2022, ‘Psychosocial barriers and facilitators for a successful return to work following injury within firefighters’, *International Archives of Occupational and Environmental Health* 95, 331–339. 10.1007/s00420-021-01712-z 33977365 PMC8795041

[CIT0041] Pelissier, C., Fort, E., Fontana, L., Charbotel, B. & Hours, M., 2017, ‘Factors associated with non-return to work in the severely injured victims 3 years after a road accident: A prospective study’, *Accident Analysis & Prevention* 106, 411–419. 10.1016/j.aap.2017.06.020 28728063

[CIT0042] Proudfoot, K., 2023, ‘Inductive/deductive hybrid thematic analysis in mixed methods research’, *Journal of Mixed Methods Research* 17, 308–326.

[CIT0043] Sharma, B.R., 2008, ‘Road traffic injuries: a major global public health crisis’, *Public health* 122(12), 1399–1406. 18950819 10.1016/j.puhe.2008.06.009

[CIT0044] Sjobbema, C., Van Der Mei, S., Cornelius, B., Van Der Klink, J. & Brouwer, S., 2018, ‘Exploring participatory behaviour of disability benefit claimants from an insurance physician’s perspective’, *Disability and Rehabilitation* 40, 1943–1952. 10.1080/09638288.2017.1323024 28490204

[CIT0045] Soeker, S., Wegner, L. & Caldwell, L.L., 2014, ‘Returning individuals with mild to moderate brain injury back to work: a systematic client centered approach’, in F. Sadaka & T. Quinn (eds.), *Traumatic brain injury*, pp.373–397, Rijeka, Crotia.

[CIT0046] Stefan, S., Shanil, E., Susan, C.A.B., Wout, E.L.D.B., Thomas, Z., Gordon, H.G. et al., 2012, ‘Return to work coordination programmes for work disability: a meta-analysis of randomised controlled trials’, *PLoS One* 7, e49760. 10.1371/journal.pone.0049760 23185429 PMC3501468

[CIT0047] Tinta, N. & Kolanisi, U., 2023, ‘Overcoming barriers for people with disabilities participating in income-generating activities: A proposed development framework’, *African journal of disability* 12, p. 1133. 37065937 10.4102/ajod.v12i0.1133 PMC10091055

[CIT0048] WFOT.org, (Internet) World Federation of Occupational Therapy. 2010, http://www.wfot.org

[CIT0049] Whiteford, G., Jones, K., Rahal, C. & Suleman, A., 2018, ‘The Participatory Occupational Justice Framework as a tool for change: Three contrasting case narratives’, *Journal of Occupational Science* 25, 497–508. 10.1080/14427591.2018.1504607

[CIT0050] Wilbanks, S.R. & Ivankova, N.V., 2015, ‘Exploring factors facilitating adults with spinal cord injury rejoining the workforce: a pilot study’, *Disability and Rehabilitation* 37, 739–749. 10.3109/09638288.2014.938177 25003483

[CIT0051] Wilcock, A.A., *An Occupational Perspective of Health*, 2nd edition. Thorofare, NJ: Slack Inc; 2006.

[CIT0052] World Health Organization, 2020, *WHO establishes Council on the Economics of Health for All*, WHO, viewed 18 November 2020, from https://www.who.int/news/item/13-11-2020-who-establishes-council-on-the-economics-of-health-for-all.

[CIT0053] World Health Organization, 2023, *Road traffic injuries*, WHO, viewed 08 September 2023, from https://www.who.int/news-room/fact-sheets/detail/road-traffic-injuries.

